# Improvement of BCG protective efficacy with a novel chimpanzee adenovirus and a modified vaccinia Ankara virus both expressing Ag85A

**DOI:** 10.1016/j.vaccine.2015.10.017

**Published:** 2015-11-27

**Authors:** E. Stylianou, K.L. Griffiths, H.C. Poyntz, R. Harrington-Kandt, M.D. Dicks, L. Stockdale, G. Betts, H. McShane

**Affiliations:** The Jenner Institute, University of Oxford, United Kingdom

**Keywords:** Tuberculosis, BCG, Vaccine, Intranasal, Viral vector, Protection, Immunogenicity

## Abstract

A replication-deficient chimpanzee adenovirus expressing Ag85A (ChAdOx1.85A) was assessed, both alone and in combination with modified vaccinia Ankara also expressing Ag85A (MVA85A), for its immunogenicity and protective efficacy against a *Mycobacterium tuberculosis* (*M.tb*) challenge in mice. Naïve and BCG-primed mice were vaccinated or boosted with ChAdOx1.85A and MVA85A in different combinations. Although intranasally administered ChAdOx1.85A induced strong immune responses in the lungs, it failed to consistently protect against aerosol *M.tb* challenge. In contrast, ChAdOx1.85A followed by MVA85A administered either mucosally or systemically, induced strong immune responses and was able to improve the protective efficacy of BCG. This vaccination regime has consistently shown superior protection over BCG alone and should be evaluated further.

## Highlights

• Intranasal immunisation with ChAdOx1.85A induces strong T-cell responses.
• ChAdOx1.85A boosted with MVA85A significantly improves the protective efficacy of BCG.
• MVA85A boost is protective both after mucosal and systemic administration.

## 1 Introduction

Tuberculosis is a global health problem, with an estimated 9 million new cases and 1.5 million deaths in 2013 [1]. BCG is the only available vaccine, and although protective against disseminated forms of childhood disease, it is not consistently protective against adult pulmonary TB [2].

Vaccine development efforts are focused on either replacing BCG (live vaccines) or identifying ‘BCG boosting’ vaccines to improve protective immunity [3]. There two live vaccines currently in clinical trials, a recombinant BCG (rBCG VPM1002) [4] and a double deletion mutant of *M.tb* (MTBVAC) [5]. Subunit vaccines are administered after BCG and focus immunity to a single or a few immunodominant antigens. Subunit vaccine antigens are delivered as either adjuvanted protein [6], [7], [8], [9] or in viral vectors. There are currently seven subunit candidates being evaluated in clinical trials [10].

Recombinant viral vectors represent a highly potent antigen delivery system and several are currently being evaluated in TB vaccine trials. The most advanced are the replication-deficient modified Vaccinia virus Ankara expressing Ag85A; MVA85A [11], and human adenovirus, AdHu35 expressing Ag85A, Ag85B and TB10.4; Aeras402 [12]. In addition, human adenovirus type 5 expressing Ag85A; AdHu5.85A, is at earlier stages of clinical testing and completed a phase I study [13].

As TB is primarily initiated by the inhalation of aerosol droplets containing *Mycobacterium tuberculosis* (*M.tb*) establishing an infection in the lung, a vaccine that induces a local lung immune response could be advantageous. Induction of lung immune responses by vaccination could potentially prevent the establishment of infection [14]. AdHu5.85A and MVA85A have both been shown to be more protective when administered by the intranasal compared to the systemic route in mice [15], [16]. In addition, MVA85A administered by aerosol in BCG-vaccinated healthy adults, was well tolerated and induced strong mucosal and systemic immune responses [17].

In this pre-clinical study we have used a replication-deficient chimpanzee adenovirus vector developed in Oxford (ChAdOx1) [18]. Unlike human adenoviral vectors, chimpanzee adenoviral vectors are not affected by pre-existing anti-vector immunity caused by previous vector exposure [19], [20]. This pre-existing immunity could reduce potency when the vaccine is administered in the human population [21], [22].

MVA85A induces primarily CD4+ T cell responses [17], [23] and recombinant adenoviral vectors induce primarily CD8+ T cell responses [24]. We used ChAdOx1, modified to express 85A, ChAdOx1.85A, alone and in combination with MVA85A to optimise both immunogenicity and protective efficacy.

## 2 Materials and methods

### 2.1 Vaccinations

Six to eight week old female Balb/c mice were purchased from Harlan, UK. All procedures were performed in accordance with the UK Animals (Scientific Procedures) Act 1986 under project licence number 30/2889 granted by the UK Home Office.

Development of E1–E3 deleted replication deficient AdHu5 and ChAdOx1 [18] to express codon-optimised Ag85A antigen from *Mycobacterium tuberculosis* has been previously described [18], [25]. Mice were vaccinated with a dose of 1 × 10^8^ infectious units (ifu) of ChAdOx1.85A and/or 5 × 10^6^ plaque forming units (pfu) of MVA85A. Vaccinations were performed via the intradermal (*i.d.*) or intranasal (*i.n.*) route, in a final volume of 50 μl.

BCG Pasteur was grown in-house in 7H9 Broth (BD, UK) containing 0.05% Tween 80. Mice were vaccinated with 4 × 10^5^ colony forming units (CFU)/dose via the *i.d.* route.

### 2.2 Challenge experiments

Mice were challenged using a Biaera AeroMP-controlled nebuliser (Biera technologies; Hagerstown, USA) contained in a Biosafety level 3 TCOL isolator. Animals were loaded in nose-only restrainers and exposed to aerosolised *M.tb* Erdman K01 (TMC107) (BEI resources; Manassas USA), prepared at 1 × 10^6^ CFU/ml in the nebuliser. The programme was run for 10 min (plus 5 min purge), airflow 12 L/min, and pressure 20 psig. Mice were infected with 50–100 CFU, verified 24 h after challenge in two mice/experiment.

### 2.3 Quantification of CFU

Lungs and spleens of infected animals were harvested four weeks after challenge. Organs were homogenised in re-inforced tubes with ceramic beads containing 1 ml PBS using Precellys 24 (Stretton Scientific, UK). Homogenised organs were diluted in PBS and dilutions were plated in Middlebrook 7H10 plates (Sigma–Aldrich), containing OADC (BD Diagnostic Systems). Plates were incubated at 37 °C and counted three weeks later.

### 2.4 Flow cytometry

Cells were extracted from the bronchoalveolar lavage (BAL) fluid, lung and spleen. BAL fluid was obtained by three successive lung lavages with 0.5 ml of 10 mM EDTA/PBS (Sigma). Lungs were perfused with PBS, chopped into small pieces, and digested in DNase/collagenase (Sigma).

Cells were stimulated with 2 μg/ml of each Ag85A peptide in a pool of 66 peptides spanning the whole sequence (or media only for unstimulated controls) and incubated for 2 h at 37 °C. Golgi plug (1 μl/ml) (BD Biosciences) was added in each well and incubated for a further 4 h followed by incubation overnight at 4 °C. The following day intracellular staining was performed.

Initially, cells were stained for 10 min with live/dead fixable dead cell stain (Invitrogen, UK) followed by surface staining with anti-CD45R/B220, TCRαβ, TCRγδ, CD4 and CD8 (eBioscience). Following permeabilisation using CytoFix/CytoPerm (BD Biosciences), cells were stained intracellularly with anti-IFN-γ, TNF-α, IL-2 and IL-17 (eBioscience). Samples were run on an LSR II flow cytometer and the data was analysed using FlowJo (TreeStar Inc, Ashland, US) and Spice 5.3 (NIAID, US).

### 2.5 Statistical analyses and presentation

Statistical analysis was conducted and graphs were generated using GraphPad Prism 5. Analysis of two data sets was performed using Mann–Whitney or Kruskal–Wallis (non-parametric) or one-way Anova followed by post hoc tests for comparing three or more groups.

## 3 Results

### 3.1 Construction of ChAdOx1 expressing Ag85A and in vivo assessment

**Fig. 1:**
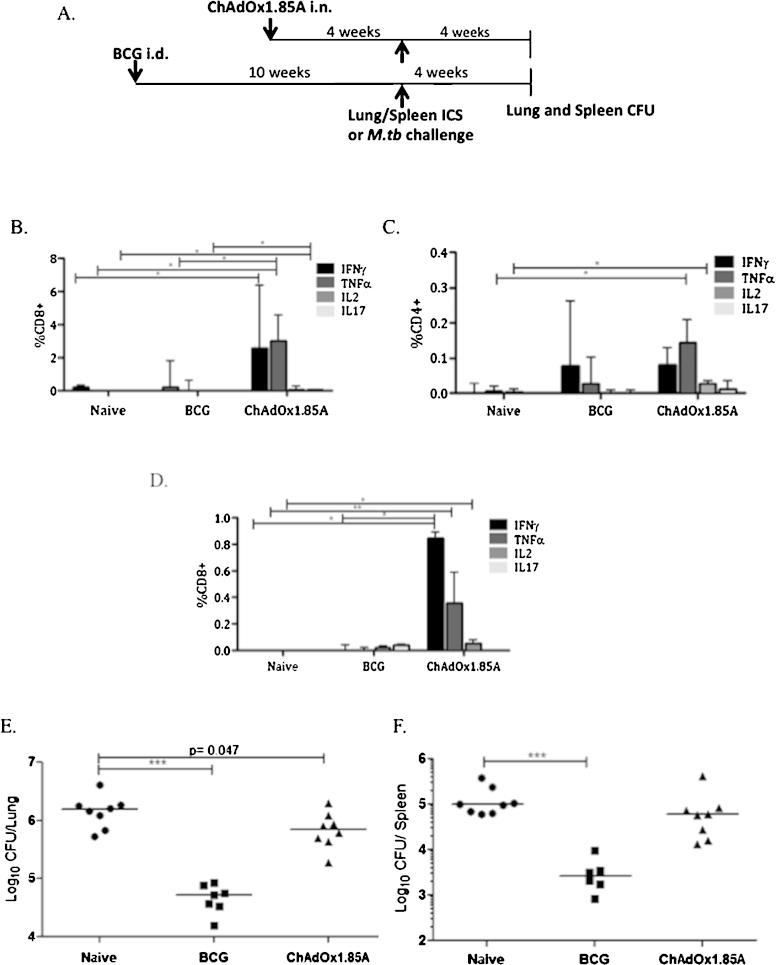
Assessing immunogenicity and protective potential of intranasally administered ChAdOx1.85A. (A) Experimental schema. (B) Balb/c mice were immunised *i.n.* with 1 × 10^8^ ifu of ChAdOx1.85A and harvested four weeks later. Percentage of CD8+ and (C) CD4+ cells secreting IFNγ, TNFα, IL2 and IL17 by lung cells. (D) Spleen CD8+ cytokine responses. (E) Lung and (F) spleen bacterial load after aerosol *M.tb* infection four weeks after the last immunisation (6 weeks after BCG). *p < 0.05, **p < 0.01.

A replication-deficient chimpanzee adenovirus previously developed in Oxford was modified to express Ag85A [25]. As mice have no pre-existing immunity to chimpanzee adenoviruses [26], they were used to assess ChAdOx1.85A vaccine potency. After a single intranasal immunisation with ChAdOx1.85A, lungs and spleens were harvested four weeks later to evaluate antigen-specific immune responses (Fig. 1A). In the lungs, ChAdOx1.85A induced a significant frequency of Ag85A-specific CD8+ T cells secreting IFNγ compared to naïve mice (*p* < 0.05) and higher TNFα and IL17 compared to both naïve and BCG-vaccinated groups (*p* < 0.05) (Fig. 1B). The percentages of cytokine-secreting CD4+ cells induced were much lower, with only TNFα and IL2 significantly higher in the vaccinated compared to naïve and BCG control group (*p* < 0.05) (Fig. 1C). In the spleen, ChAdOx1.85A induced systemic CD8+ cells secreting IFNγ, TNFα and IL2 (Fig. 1D). CD4+ cells secreting cytokines were very low (data not shown).

Four weeks after ChAdOx1.85A administration, mice were challenged with *M.tb* via the aerosol route and harvested four weeks later for lung and spleen bacterial enumeration (Fig. 1A). BCG-vaccinated animals had significantly lower bacterial load compared to unvaccinated mice (reduced by 1.46 log_10_ in lung, *p* < 0.001 and 1.57 log_10_ in spleen, *p* < 0.001) whereas ChAdOx1.85A only slightly decreased the bacterial load of vaccinated compared to unvaccinated mice in the lung (0.32 log_10_, *p* = 0.047,) and spleen (0.2 log_10_, *p* = 0.08) (Fig. 1E and F).

### 3.2 Boosting ChAdOx1.85A with MVA85A, optimising the time interval

**Fig. 2:**
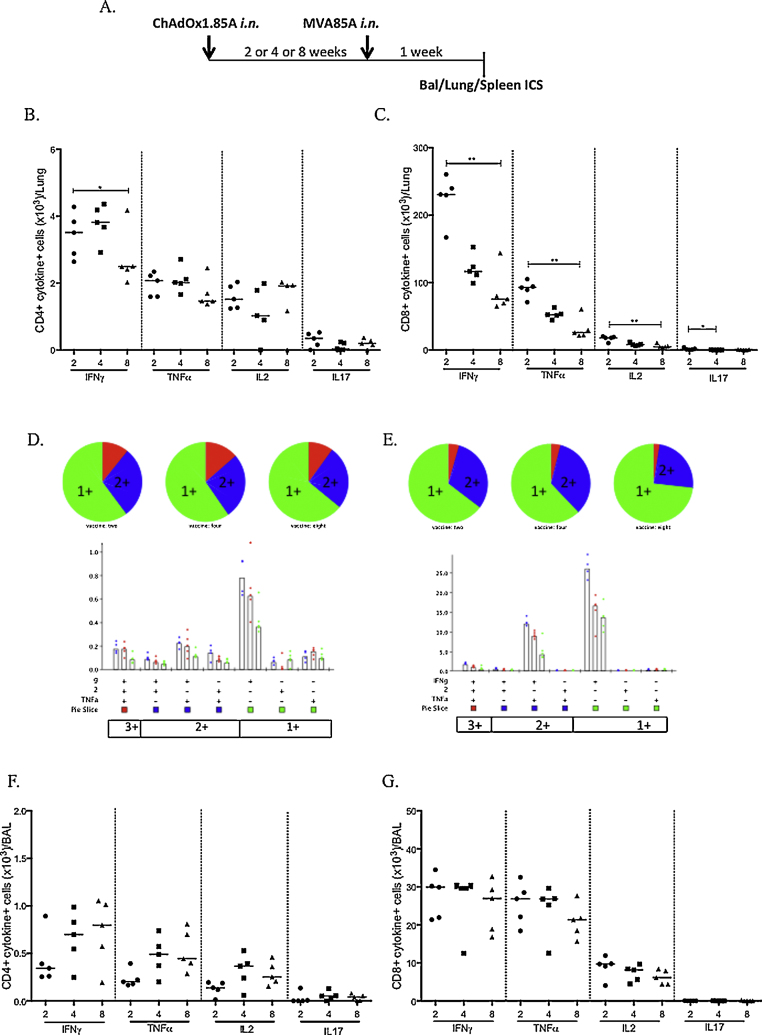
Optimisation of the time interval between intranasal ChAdOx1.85A and intranasal MVA85A. Lung and BAL data presented. (A) Experimental schema. Absolute numbers of lung CD4+ cells (B) and CD8+ cells (C) secreting different cytokines at two, four or eight week intervals between the two vaccinations. Cytokine responses were measured one week after the MVA85A vaccination. (D) Pie chart and bar chart showing the polyfunctionality of the CD4+ cells. Bar chart: blue dots represent 2, red dots and green dots the 8 week interval. (E) Pie chart and bar chart showing the polyfunctionality of the CD8 cells. (F) Absolute numbers of BAL CD4+ and (G) CD8+ T cell populations secreting different cytokines one week after MVA85A vaccinations.

Since ChAdOx1.85A induced strong CD8+ T cell responses we decided to use MVA85A, a strong inducer of CD4+ responses, as a booster vaccine. To identify the optimal time interval between the two vaccinations, mice were vaccinated *i.n.* with ChAdOx.85A and boosted *i.n.* with MVA85A 2, 4 or 8 weeks later. One week after the MVA85A vaccination, BAL, lung and spleen were collected from each mouse to measure Th1 immune responses (Fig. 2A).

In the lungs, there was a significantly higher number of CD4+ IFNγ producing cells in the 2 compared to the 8 week interval (*p* < 0.05) (Fig. 2B). CD8+ responses were significantly higher in the 2 week compared to the 8 week interval for IFNγ (*p* < 0.01), TNFα (*p* < 0.01) and IL2 (*p* < 0.01) (Fig. 2C). The populations of cytokine-secreting cells were mainly single or double cytokine producers for both CD4+ and CD8+ T cells (Fig. 2D and E). There were no differences in the number of cytokine secreting CD4+ or CD8+ cells in the BAL between groups (Fig. 2F and G), whereas spleen responses were lower than the BAL and lung responses, with the 4 week interval inducing stronger CD8+ IFNγ responses (data not shown).

Based on the above data we decided to proceed with the 4 week interval between ChAdOx.85A and MVA85A.

#### 3.3.1 Optimisation of vaccination regime: immunogenicity studies

**Supplementary Figure 1:**
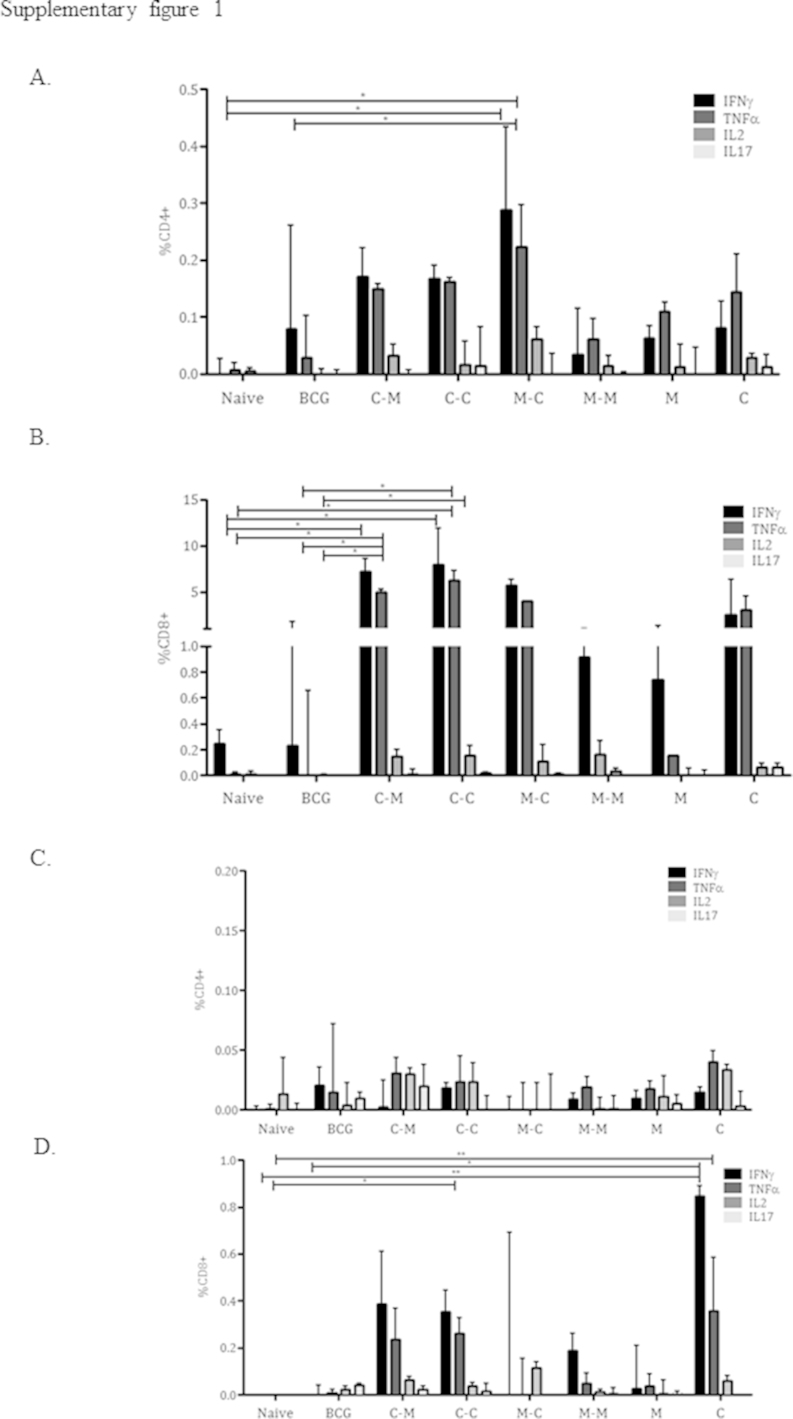
Additional data for Fig. 3, showing different cytokines secreted by CD4+ and CD8+ cells in lung (A and B) and spleen (C and D).

**Fig. 3:**
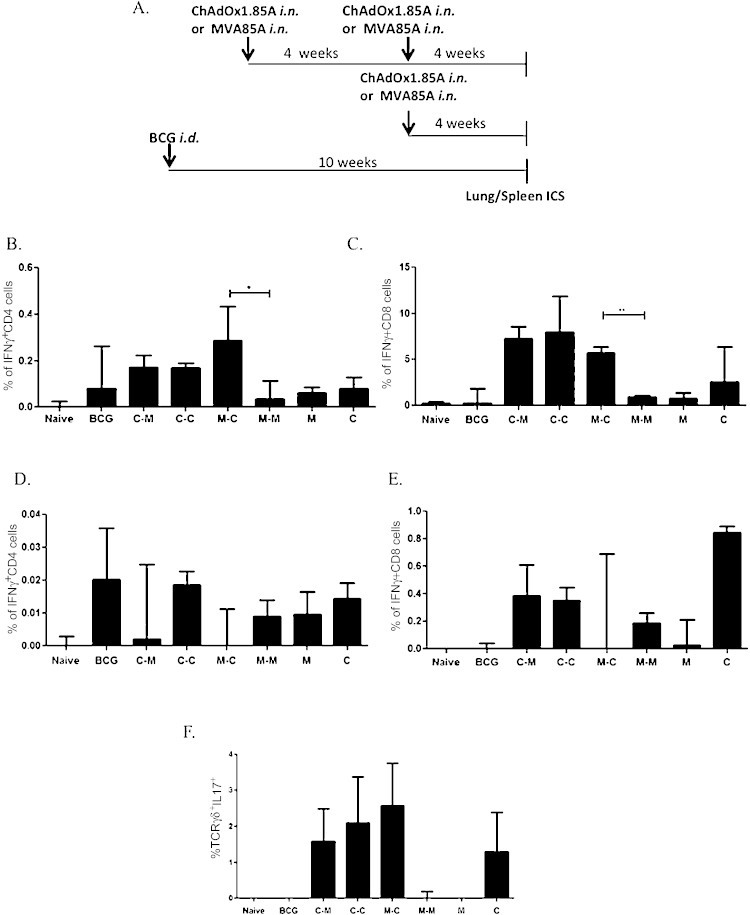
Optimisation of the vaccination regime with homologous or heterologous prime boost vaccinations. (A) Experimental schema. (B and C) Lung CD4+ and CD8+ IFNγ secreting T cells detected in the lungs of mice four weeks after the last vaccination. (D and E) Spleen CD4+ and CD8+ IFNγ secreting T cells detected four weeks after the last vaccination. (F) TCRγδ IL17 secreting T cells in the lungs of vaccinated animals.

We then optimised a vaccination regime based on the two viral vectors. Balb/c mice were either homologously or heterologously boosted with ChAdOx1.85A (C) or MVA85A (M) via the *i.n.* route. Three control groups ChAdOx1.85A *i.n.* alone, MVA85A *i.n.* alone and BCG *i.d.* alone were included. Four weeks after the last immunisation, lung and spleen responses were evaluated using ICS assay (Fig. 3A). All regimes were able to induce CD4+ and CD8+ T cells in the lungs. The M–C regimen was optimal at inducing CD4+ IFNγ and TNFα and the C–C and C–M regimen was optimal for the induction of CD8+ IFNγ and TNFα (Fig. 3B and C for IFNγ and supplementary Figure 1A and B for all cytokines). No vaccine regime was able to induce strong CD4+ responses in the spleen. C alone induced the strongest systemic CD8+ IFNγ and TNFα responses; C–M and C–C also induced good CD8+ responses (Fig. 3D and E for IFNγ and supplementary Figure 1C and D for all cytokines). Mice that received ChAdOx1.85A (C–M, C–C and C) all had TCRγδ IL17 responses in the lung (Fig. 3F).

Supplementary Figure 1 related to this article can be found, in the online version, at http://dx.doi.org/10.1016/j.vaccine.2015.10.017.

#### 3.3.2 Optimisation of vaccine regime: protection studies

**Fig. 4:**
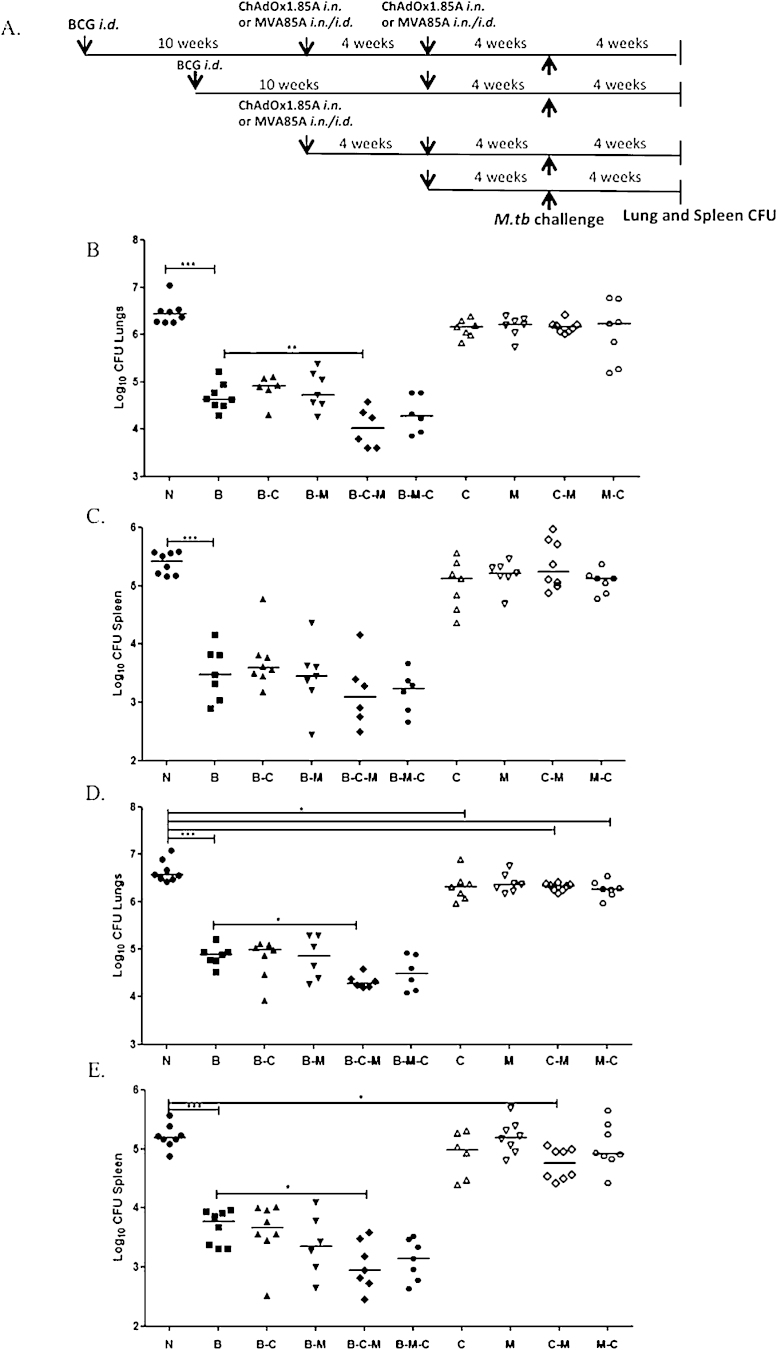
Assessing the protective potential of different vaccine combinations administered alone or as a boost to BCG. (A) Experimental schema. (B) Lung CFU data from mice four weeks after *M.tb* Erdman aerosol challenge. ChAdOx1.85A was administered *i.n.* and MVA85A *i.d.* (C) Spleen CFU data. (D) Lung CFU data from a separate challenge experiment where both ChAdOx1.85A and MVA85A were administered via the *i.n.* route. (E) Spleen CFU data. Each symbol represents one animal and the line the median CFU value of each group.

To evaluate the protective efficacy of different vaccination regimes, two duplicate challenge experiments were set up. In one experiment Balb/c mice received ChAdOx1.85A *i.n.* and MVA85A *i.d.* 4 weeks later (with and without BCG prime); and in the second experiment the MVA85A boost was administered *i.n.* with the same interval between vaccinations. Mice were challenged with aerosolised *M.tb* four weeks after the last immunisation and lung and spleen harvested four weeks later for CFU enumeration (Fig. 4A).

In the first experiment (MVA85A administered *i.d.*) all the BCG-primed groups had significantly lower bacterial load in the lungs compared to naïve mice and to mice that were not BCG primed (*p* < 0.001) (Fig. 4B). The B–C–M vaccinated mice had significantly lower lung CFU than all the other BCG primed groups (*p* < 0.01) apart from B–M–C (which was not significantly better when compared to BCG). In the spleen, all groups that were primed with BCG had lower CFU compared to naïve and non-BCG primed animals but none of the groups had lower bacteria compared to the BCG group (Fig. 4C).

When the experiment was repeated with MVA85A administered *i.n.*, C–M, M–C and C vaccinated mice had lower bacteria in the lungs (*p* < 0.05) when compared to unvaccinated mice but only C–M vaccination significantly reduced the CFU in the spleen (*p* < 0.05); BCG-primed mice had significantly lower load in both the lung and spleen if they were boosted with C–M (B–C–M group) (*p* < 0.05) (Fig. 4D and E).

### 3.4 Immunogenicity and protective efficacy of the most promising vaccination regimes

**Fig. 5:**
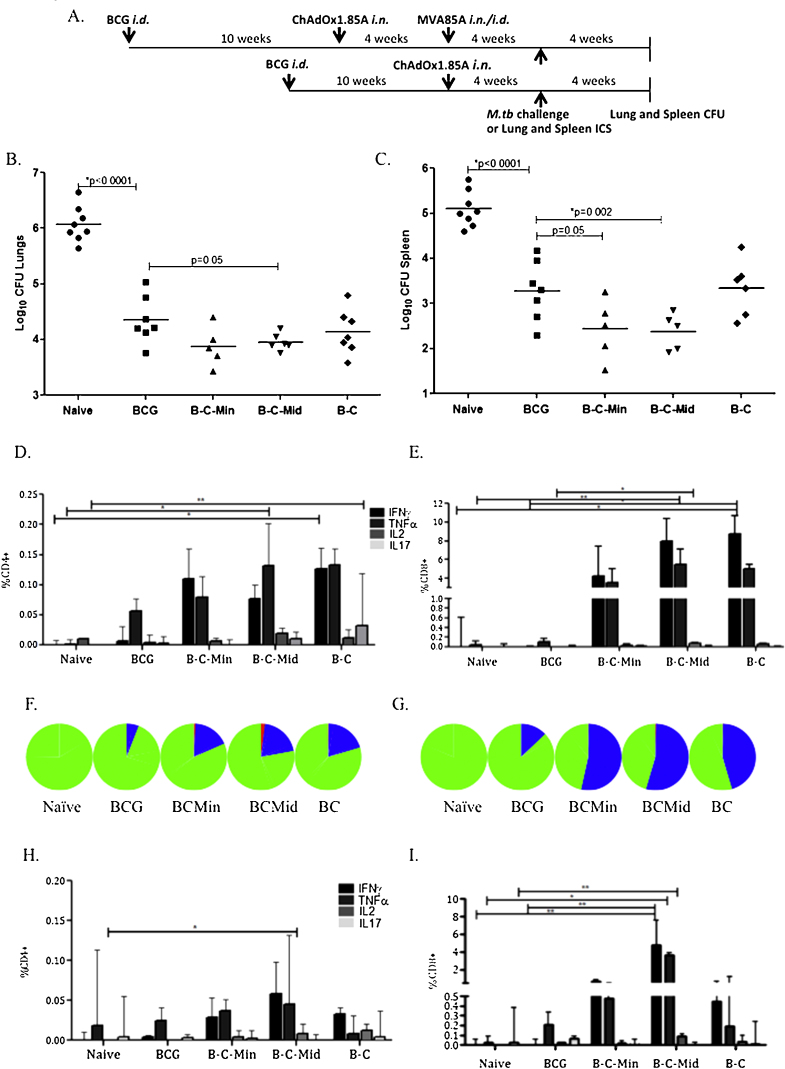
Repetition of efficacy and immunogenicity of the most promising regimes. (A) Experimental schema. (B) Mice challenged with aerosol *M.tb* Erdman four weeks after the last vaccination. Each symbol represents the lung bacterial load of one animal and the line the median of each group. (C) Spleen CFU data. Immunogenicity was measured four weeks after the last vaccination and just before challenge. ICS was performed on lung samples. Each different colour bar represents the median value of one cytokine and the bar the range of each group. (D) Results from CD4+ and (E) CD8+ T cells. (F) Polyfunctionality of CD4+ T cells, (G) polyfunctionality of CD8+ T cells. Red colour on pie chart: IFNγ, TNFα and IL2 secreting cells, blue: double, green: single cytokine secreting cells. (H) Percentage of CD4+ and (I) CD8+ T cells secreting cytokines in the spleen.

To directly compare the two most protective regimes, mice were vaccinated with either B–C–M (M administered *i.d.*) or B–C–M (M administered *i.n.*). Control groups included BCG alone or BCG followed by C. Protective efficacy and immunogenicity was assessed (Fig. 5A).

The BCG-vaccinated group had significantly lower bacterial counts compared to the naïve control group (*p* < 0.0001). In addition, C–M*i*.*d.* significantly improved the protective efficacy of BCG (*p* = 0.05 in the lungs and *p* = 0.002 in spleen) whereas C–M*i*.*n.* approached significance in the lung and was significant in the spleen (*p* = 0.07 lung, *p* = 0.05 spleen) (Fig. 5B and C).

In a parallel immunogenicity experiment, Ag85A specific CD4 and CD8 cytokine responses were measured in lungs and spleen, four weeks after the last immunisation. ICS was used to quantify and compare the percentage of cytokine secreting CD4+ and CD8+ cells between groups. All BCG boosting vaccines induced stronger CD8+ than CD4+ T cells in both the lung (Fig. 5D and E) and spleen (Fig. 5H and I). BCG followed by ChAdOx1.85A was significantly better at inducing IFNγ CD8+ cells in the lung when compared to naïve and BCG-vaccinated mice. A further boost with MVA85A failed to enhance IFNγ production but *i.d.* MVA85A enhanced both TNFα and IL2 responses. Boosting with ChAdOx1.85A and MVA85A increased the double cytokine secreting CD4+ and CD8+ cells in the lungs (Fig. 5F and G). In the spleen, BCG-C–M *i.d.* induced the strongest CD8+ T cell response (Fig. 5I).

## 4 Discussion

BCG is not effective against pulmonary TB, and subunit vaccines designed to boost its immunity and protective efficacy are required. One vaccination approach is the use of replication-deficient viral vectors. These are very immunogenic and have a good safety record as evidenced by a number of clinical trials against TB and a number of other infectious diseases.

**Supplementary Figure 2:**
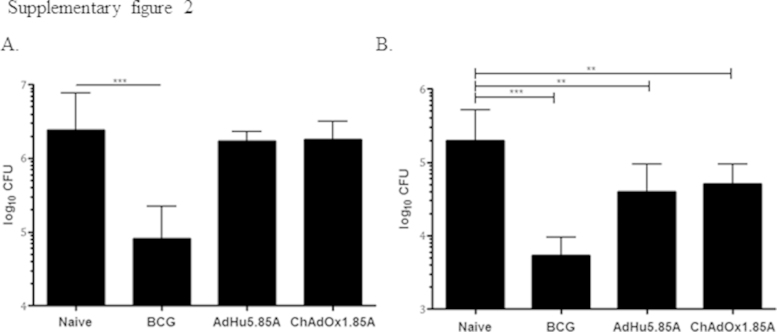
Challenge results after a single intranasal administration of 1 × 10^8^ ifu of virus. (A) Lung and (B) spleen CFU data. A group of mice was vaccinated *i.d*. with BCG and challenged 6 weeks later; in parallel, one group of mice received AdHu5.85A and another group ChAdOx1.85A, both *i.n*. and challenged four weeks later with aerosol *M.tb* Erdman. Experiment was terminated four weeks after challenge.

**Supplementary Figure 3:**
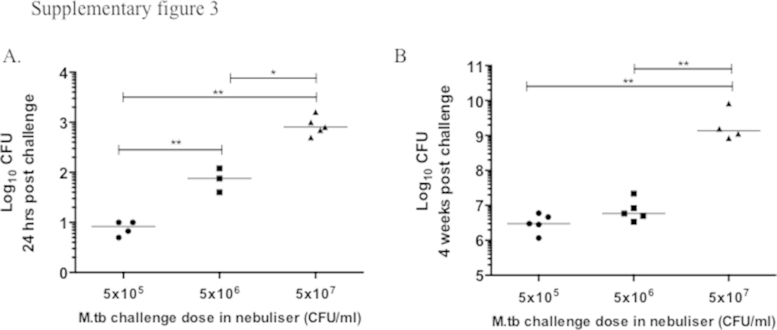
Mycobacterial dose escalation challenge experiment. (A) Some mice were harvested 24 h post infection to measure infection levels. (B) Lung bacterial load 4 weeks after infection (the group challenged with 5 × 10^7^ CFU/ml in the nebuliser had to be culled before the 4 week time point).

A single intranasal immunisation with ChAdOx1.85A, alone or as a boost to BCG was highly immunogenic, inducing strong immune responses in the lungs and spleen. However, it resulted in no or small reduction in the bacterial load of vaccinated animals (Figs. 1 and 4B–E). This absence of protection is in contrast to the intranasal administration of human adenovirus expressing the same antigen [15], [27]. In mice AdHu5.85A has previously shown superior CD8+ immunogenicity to ChAdOx1.85A, when administered intramuscularly (only in high doses) [25]. In contrast, there was no difference in protective efficacy when we directly compared *i.n.* vaccination with our AdHu5.85A versus ChAdOx1.85A, followed by aerosol *M.tb* challenge (supplementary data Figure 2). The reason for this discrepancy is not clear but a possible explanation is that our infection model is more stringent. An infection with approximately 8 CFU, measured 24 h post infection, resulted in 10^6^ CFU in the lungs of infected mice at 4 weeks post infection and a dose of 800 CFU at 24 h post infection (supplementary data Figure 3A) lead to 10^9^ CFU in the lungs and mice succumbing to disease at day 24, 4 days before the scheduled 4 week time point (supplementary data Figure 3B). In contrast, when we compare this to previously published data using aerosol challenge, the same high challenge dose (880 CFU) was not as virulent and resulted in a 10^6^ CFU lung load, 4 weeks post infection [28]. This suggests that our challenge application is particularly efficient, generating small particles, 1–2 μm (which then dry down and become smaller), which can disseminate more efficiently throughout and deep in the lungs [29]. Particle size and deposition have been shown to have an effect on pathogenicity of an organism [30]. This system is perhaps more similar to more stringent animal species e.g. the guinea pig. When AdHu5.Ag85A was used to boost BCG-vaccinated guinea pigs, there was no reduction in the bacterial load compared to BCG-vaccinated animals [31].

Supplementary Figures 2 and 3 related to this article can be found, in the online version, at http://dx.doi.org/10.1016/j.vaccine.2015.10.017.

One significant limitation of homologous prime-boost vaccination regimens with the same viral vector is the induction of anti-vector immunity induced after the primary vaccination. In this study, mucosal homologous boosting of both ChAdOx1.85A and MVA85A (Fig. 3) did not result in improvement in the lung immune responses compared to a single vaccination, in agreement with published data [32]. For this reason, heterologous prime-boost immunisations with viral vectors are more promising. A simian adenoviral vector, ChAd63, expressing malaria antigens, followed by MVA expressing the same malaria antigens, induced strong immune responses that were higher than those induced by one vector alone in clinical trials [33], [34]. Surprisingly, boosting ChAdOx1.85A with MVA85A did not induce stronger IFNγ when compared to ChAdOx1.85A followed by ChAdOx1.85A or to ChAdOx1.85A alone (the median response was higher for the first two), whereas MVA85A followed by ChAdOx1.85A was better than MVA85A followed by MVA85A (Fig. 3). Even though the level of immune response was similar between those groups it is possible that the breadth of the immune response differs and this is worth investigating further [34]. When C–M and M–C were administered *i.n.* (Fig. 4D) they were able reduce the bacterial load compared to unvaccinated mice, however only the C–M regimen was able to significantly improve BCG efficacy (Fig. 4, Fig. 5), whichever the route of MVA85A. A possible reason for this efficacy could be the ability of C–M to induce central memory responses which are not induced by BCG vaccination and might be needed for durable protection [35], [36].

B–C–M*i*.*n.* and B–C–M*i*.*d.* were directly compared in a challenge experiment, only B–C–M*i*.*d.* significantly improved BCG; although the B–C–M*i*.*n*. and B–C–M*i*.*d.* were not significantly different (Fig. 5B and C). A likely explanation for the absence of protection is that the slightly higher BCG protective efficacy obtained in the repeat experiment, which approached two log 10 CFU, improvement compared to naïve mice minimised the potential to observe a further improvement in efficacy by additional vaccinations.

Although the immune responses measured in these experiments did not correlate with protective efficacy, further work will aim to identify potential correlates of protection that are unique to the B–C–M regime. Further experiments, will address the ability of B–C–M to induce antigen specific memory responses and to provide durable protection when mice are challenged much later after vaccination. In addition, the distribution of antigen-specific T cells in different lung compartments will be further explored [37].

ChAdOx1 expressing influenza A antigens was safe and immunogenic in clinical trials [38] and ChAdOx1.85A, currently being evaluated in a phase I first-time-in-man study is also showing good safety and immunogenicity (McShane, personal communication), making this a promising vaccine candidate. This study suggests that a further boost with MVA85A could potentially improve its immunogenicity and its protective efficacy. In the malaria field, in a clinical challenge study, heterologous prime boost with ChAd63 followed by MVA, both expressing METRAP, conferred 21% efficacy, in contrast to control volunteers and individuals vaccinated with ChAd63-METRAP, who all developed malaria [39].

ChAdOx1.85A followed by intranasal or intradermal MVA85A as a boost to BCG is a promising vaccination regimen that should be investigated further for protective efficacy in other preclinical *M.tb* infection models. A recent study using ChAdOx1.85A was very immunogenic in cattle [25]; it would therefore be interesting to see whether this can be improved further with MVA85A and more importantly whether this also correlates with protective efficacy.
